# From diet to function: using synthetic microbial communities to map gut microbial interactions

**DOI:** 10.1038/s41522-026-01012-9

**Published:** 2026-06-15

**Authors:** Dodzi Kwaku Jnr. Senoo, Luke Acton, Lindsay J. Hall

**Affiliations:** 1 Department of Microbes, Infection and Microbiomes, School of Infection, Inflammation and Immunology, College of Medicine and Health, University of Birmingham, Birmingham, UK; 2 Institute of Microbiology and Infection, University of Birmingham, Birmingham, UK; 3 Food, Microbiome, & Health, Quadram Institute Bioscience, Norwich Research Park, Norwich, UK; 4 Norwich Medical School, University of East Anglia, Norwich, UK

**Keywords:** Biological techniques, Computational biology and bioinformatics, Microbiology, Systems biology

## Abstract

Diet shapes gut microbial ecology and function, but causal mechanisms are hard to resolve in native communities. Synthetic microbial communities provide defined systems to test how dietary substrates alter community structure, metabolism, and host responses. This review summarises SynCom design, experimental platforms, and dietary applications, highlights multi-omics and modelling approaches, proposes reporting standards, and considers challenges and future directions, including multi-kingdom and AI-enabled function-first SynComs.

## Introduction

The human gut microbiota comprises a diverse community of microorganisms that live symbiotically with their host. This microbial ecosystem supports health through digestion of complex carbohydrates, biosynthesis of vitamins, immune modulation, Short Chain Fatty Acids (SCFAs) production, neurodevelopment, and colonisation resistance against pathogens^1–3^.

Its composition varies markedly across age, ethnicity, sex, and lifestyle^4–7^. In infants, gestational age (preterm/full term), delivery mode (vaginally/caesarean), feeding (human milk/formula), and antibiotics shape early assembly^7–10^. In adults, diet, geography, host genetics/physiology, and medications are key drivers^11–14^. Among these factors, diet is a principal modulator: macronutrient availability, fermentable fibre, and bioactive compounds can rapidly and persistently remodel microbial ecology^15–18^.

Disentangling causality in such a complex ecosystem is challenging. Synthetic microbial communities (SynComs); defined cultivable consortia of gut microorganisms, offer a controlled and reproducible approach to test how specific substrates and nutritional patterns reshape community structure and function and, in host associated models, impact physiology^19–21^. Advances in culturomics - high-throughput bacterial recovery approaches that employ multiple growth media and varied culture conditions combined with rapid identification to maximise the recovery and characterisation of diverse microorganisms from complex samples, anaerobic culturing, in vitro fermentation systems, gnotobiotic models, and multi-omics now enable the construction and mechanistic interrogation of SynComs at increasing ecological depth^22–26^.

This review outlines core principles for designing and building SynComs to study diet-microbiota interactions. We summarise experimental models used to investigate nutritional effects and microbial cross-feeding and discuss how specific dietary components shape SynCom ecology and metabolism. We also highlight analytical approaches, key applications, and practical challenges for reproducible SynCom research. Although we focus primarily on gut-associated SynComs, the design principles are broadly applicable across diverse ecological niches.

### SynComs: rationale, types, and design principles

#### Why SynComs?

SynComs offer unique mechanistic insight into microbiome function by enabling controlled, reproducible experiments that are rarely achievable in traditional studies of complex natural communities. SynComs provide a reductionist, controllable framework that overcomes key limitations of observational designs, namely high community complexity, host and ecological variability, and limited causal inference^27–29^. By precisely specifying community composition, environmental inputs, and dietary variables, SynComs support stepwise addition or removal of taxa, controlled dietary perturbations, and replication under identical conditions, allowing direct attribution of metabolic and functional outputs to defined members or interactions. When combined with host‑associated models, they also disentangle microbial from host contributions, strengthening causal interpretation^19,27,30,31^.

Overall, SynComs reduce native complexity while preserving representative taxa and metabolic capabilities, enabling: (i) causal tests of diet-microbiota (and depending on the model also host) relationships; (ii) identification of functions (e.g. SCFA production, mucin/HMO degradation, pathogen inhibition) to specific members; and (iii) evaluation of dietary, prebiotic/probiotic, and pharmacological interventions in a defined microbial background^19,21,32^.

#### Types of SynComs

SynComs can differ widely in both structural complexity (the number and types of species/strains included) and in the resolution of characterisation, ranging from broadly defined taxa to fully genome sequenced, strain-resolved members^21^ (see Table 1). Simple SynComs typically include 2-10 organisms and are designed to test specific interactions, responses, or metabolic pathways^32^. More complex SynComs incorporate a wider range of taxa to better approximate gut community structure while maintaining full control over membership composition^21,32^. SynComs also vary by how completely members are characterised. Fully defined SynComs consist only of genomically characterised strains, enabling direct links between genotype and community function, whereas semi-defined SynComs combine well characterised isolates with partially characterised members to balance experimental control with ecological realism^21,33^. Ultimately, SynCom choice depends on the research question: simpler communities may suit in-depth mechanistic studies, while more complex SynComs are potentially better for modelling community-level behaviour and interactions^21,34,35^.

**Table 1:** Classifications of SynComs: descriptions, advantages and limitations

Type	Sub-Type	Description	Advantages	Limitations	References
Simple SynComs	Fully-defined	Low complexity community typically made up of 2– 10 cultured and genomically characterised species/strains to test specific mechanistic hypotheses	Easy to setup; Reproducible; Mechanistic clarity. Ease of standardisation. Lower cost	Limited ecological realism; May miss functional redundancy; strong bias towards cultivable organisms	^175,176^
Complex SynComs	Fully-defined	High- complexity communities ( > 10–100 species/strains) composed exclusively of cultured and genomically profiled strains simulating native structure/function	Higher ecological relevance; Captures cross-feeding and competition; High reproducibility relative to semi-defined systems.	Costly; Strong bias towards culturable organisms; May still incompletely mimic native microbiota.	^29,177^
Complex SynComs	Semi-defined	Microbial communities consisting of a defined core of cultured species/strains supplemented with undefined or partially characterised inocula (e.g. faecal matter)	Improved ecological realism; Includes difficult to culture taxa;	Lower reproducibility; compositional uncertainty	^31,178^

### Selecting strains

Selecting microbial strains is a critical step in building SynComs because it determines both ecological validity and functional relevance. Strains are typically chosen for their ecological importance, metabolic capabilities (needed for diet-microbe SynComs), and how well they represent the gut microbiota of the relevant life stage or physiological condition^36^. Together, these criteria ensure SynComs capture key aspects of microbiome diversity and function in a controlled system.

#### Sources of bacteria isolates

Bacterial isolates used in SynComs are typically sourced from faecal samples or from established culture collections^37,38^. Isolating strains from faeces collected from individuals at the relevant age or physiological state can yield ecologically adapted strains that better reflect real world host-microbe and microbe-microbe interactions^37^, or from other gut niches such as the small intestine, which may harbour distinct microbial populations with unique capabilities or host interactions that are relevant to the experimental question^39^. Selecting strains from a single donor or a naturally stable ecological niche where microorganisms are already known to coexist may enhance ecological compatibility and improve stability in the SynCom. In contrast, culture collections provide strains that are often already genotypically and phenotypically characterised^40^, supporting reproducibility and enabling detailed functional studies, particularly when building fully defined SynComs.

#### Ecological relevance and functional capacity

The human gut microbiota evolves from early life to adulthood^41^, and is shaped largely by diet^42^. Strain selection must therefore reflect both the age and dietary context of the target population.

In early life, the gut microbiota of human-milk fed infants is dominated by *Bifidobacterium* spp., which can utilise complex sugars found in human milk, i.e. Human Milk Oligosaccharides (HMOs)^43^. SynComs that model the human milk-fed infant gut should therefore include HMO-adapted strains to capture these core early life metabolic functions, noting that microbial composition and functional capacity may differ in formula-fed infants. As solid foods are introduced, the microbiota diversifies and shifts towards taxa that can degrade complex plant fibres^44^. SynComs representing later life stages should therefore incorporate primary fibre degraders to reflect this dietary transition.

Each strain’s metabolic capacity (e.g., CAZymes (Carbohydrate Active enZymes - group of enzymes that build, breakdown or modify complex carbohydrates); butyrogenesis; bile acid transformations; amino‑acid fermentation), and interaction potentials (e.g. cross‑feeding, acid/bacteriocin production, type VI secretion systems (T6SS), inducible prophages) must also be evaluated. To further strengthen the ecological framework, SynCom design should also consider strategies to enhance community stability and coexistence. This includes metabolic niche partitioning, where strains are selected to occupy complementary resource niches reducing direct competition; the incorporation of ecological redundancy - ensuring multiple taxa in the SynCom can perform similar functions to buffer against perturbations^19,21,29^.

These are important considerations to ensure SynComs capture not only microbial community composition but also functional activity, while minimising competitive exclusion that can cause loss of members and distort the biochemical outputs typical of the native gut microbiota.

**Fig. 1:**
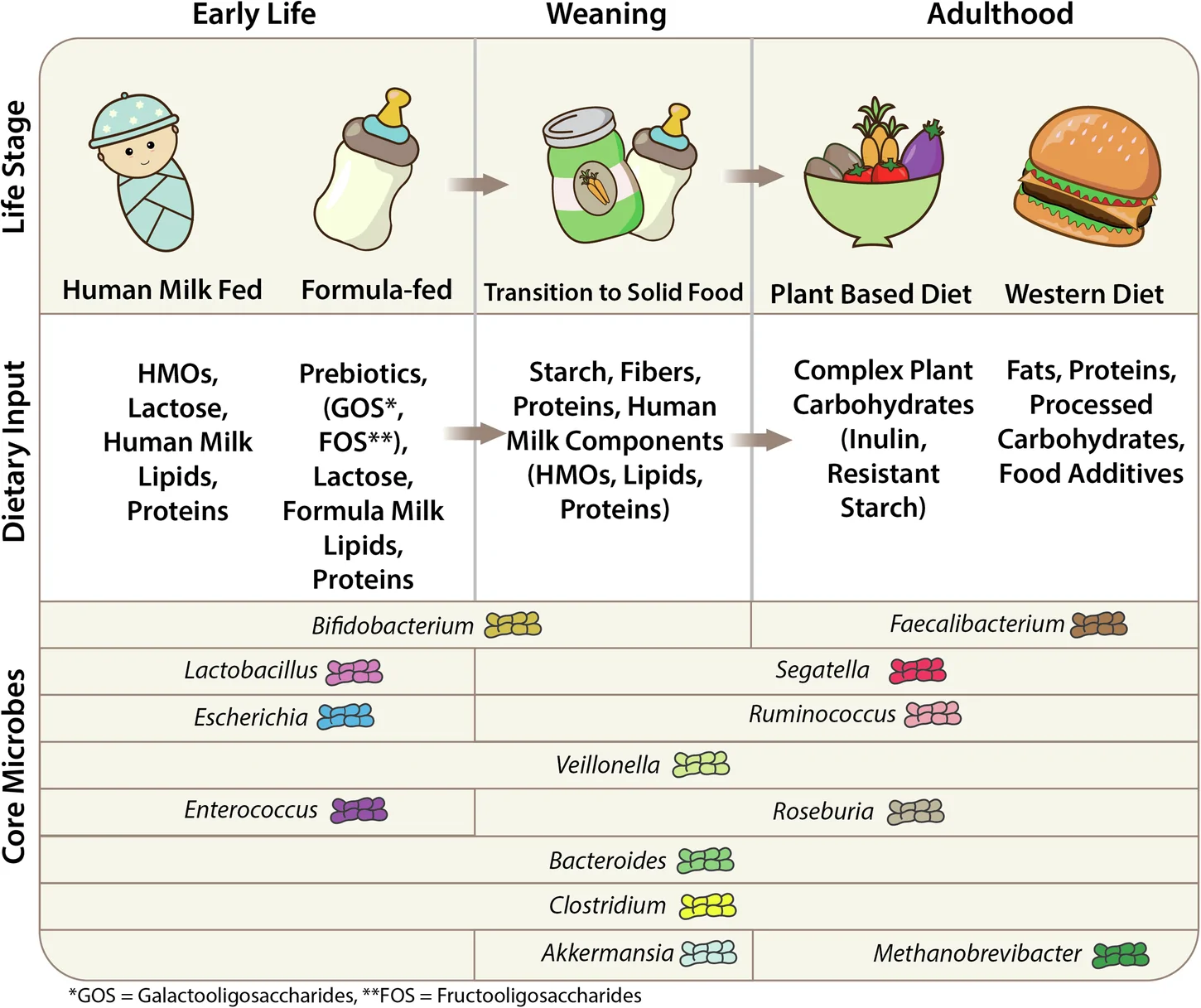
SynComs design across life with dietary input^83,191^.

Strain selection therefore must integrate ecological origin, life-stage, dietary context, and functional capacity. A conceptual framework of SynCom design across life course aligned with dietary transitions is illustrated in Fig. 1.

### Experimental models for studying SynComs

SynComs research depends on experimental models that capture key features of the gut’s ecological and physiological environment, while allowing tight control over experimental conditions^21^. Broadly, these approaches fall into in vitro and host-associated systems^45,46^.

#### In vitro models

Batch fermentations are closed, short-term anaerobic cultures that are well suited for rapid, low-cost testing of growth kinetics, basic metabolite outputs, and preliminary microbe-microbe interactions^26,47–49^. They are widely used for complex inputs (e.g., faecal slurries), but can also be readily adapted for SynComs. Their main limitation is that nutrient depletion and metabolite build-up reduce stability over longer time frames.

**Fig. 2:**
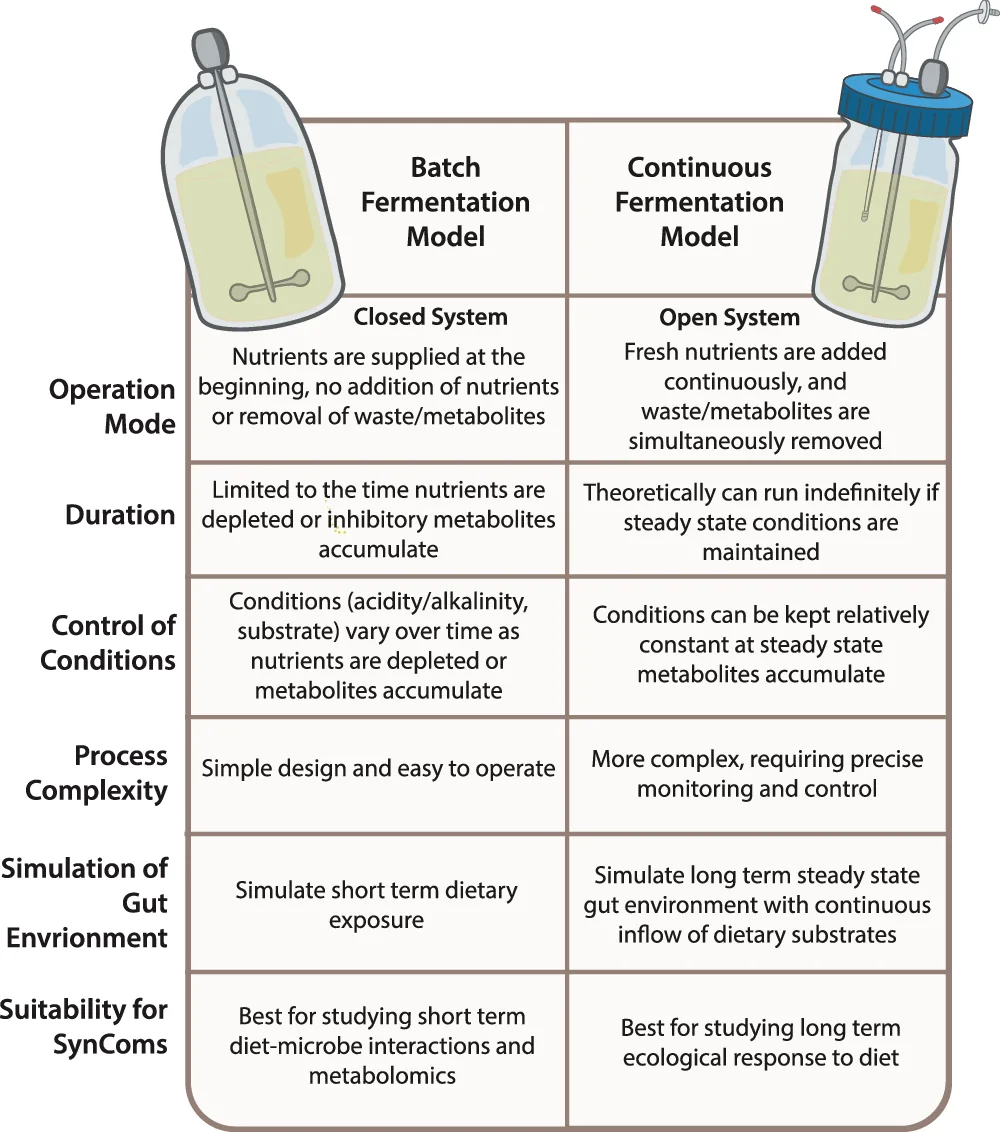
Comparison of Batch and Continuous Fermentation modes in SynCom Studies^82,192–197^.

Chemostats and more advanced dynamic gut models, such Simulator of the human Microbial Intestinal Ecosystem (SHIME) or M-SHIME (includes mucin beads to models the mucosal-associated microbiota), Toddler Mucosal Artificial colon (Tm-ARCOL), Polyfermenter Intestinal Model (PolyFermS), Human-Microbal Crosstalk model (HuMiX)/gut-on-a-chip, micro-Matrix bioreactor platforms (OPTIMATRIX v2.0) and the TNO in vitro model of the colon (TIM-2), address these constraints by providing continuous nutrient supply and waste removal, supporting steady-state growth and more physiologically realistic conditions^50–56^. These platforms can mimic gut physicochemical parameters, transit, and mucosal surfaces, supporting longer-term studies of microbial cross-feeding, dietary perturbations, and community stability, whether using faecal communities or defined SynComs^57–59^, and a summarised comparison of batch fermentation vs. continuous fermentation models is presented in Fig. 2.

#### Host-associated models

Gnotobiotic mice and non-murine models such as pigs, bees, zebrafish, and *Drosophila*, colonised with defined SynComs enable direct assessment of host outputs, including immune, metabolic, and barrier responses^19,60–66^. The development of minimal defined consortia (e.g., OMM12)^61,62^ and the use of harmonised gnotobiotic protocols such as National Gnotobiotic Rodent Resource Center (NGRRC)^67^ are improving reproducibility and cross-study comparability. These approaches provide deep insights into developmental programming, immune modulation, epithelial barrier regulation and metabolic impacts^68–71^.

Together, in vitro and host-associated systems provide a practical continuum, from controlled mechanistic testing to integrated host–microbe biology, supporting more robust inference about gut microbial ecology and function.

### Dietary modulation of SynComs

**Fig. 3:**
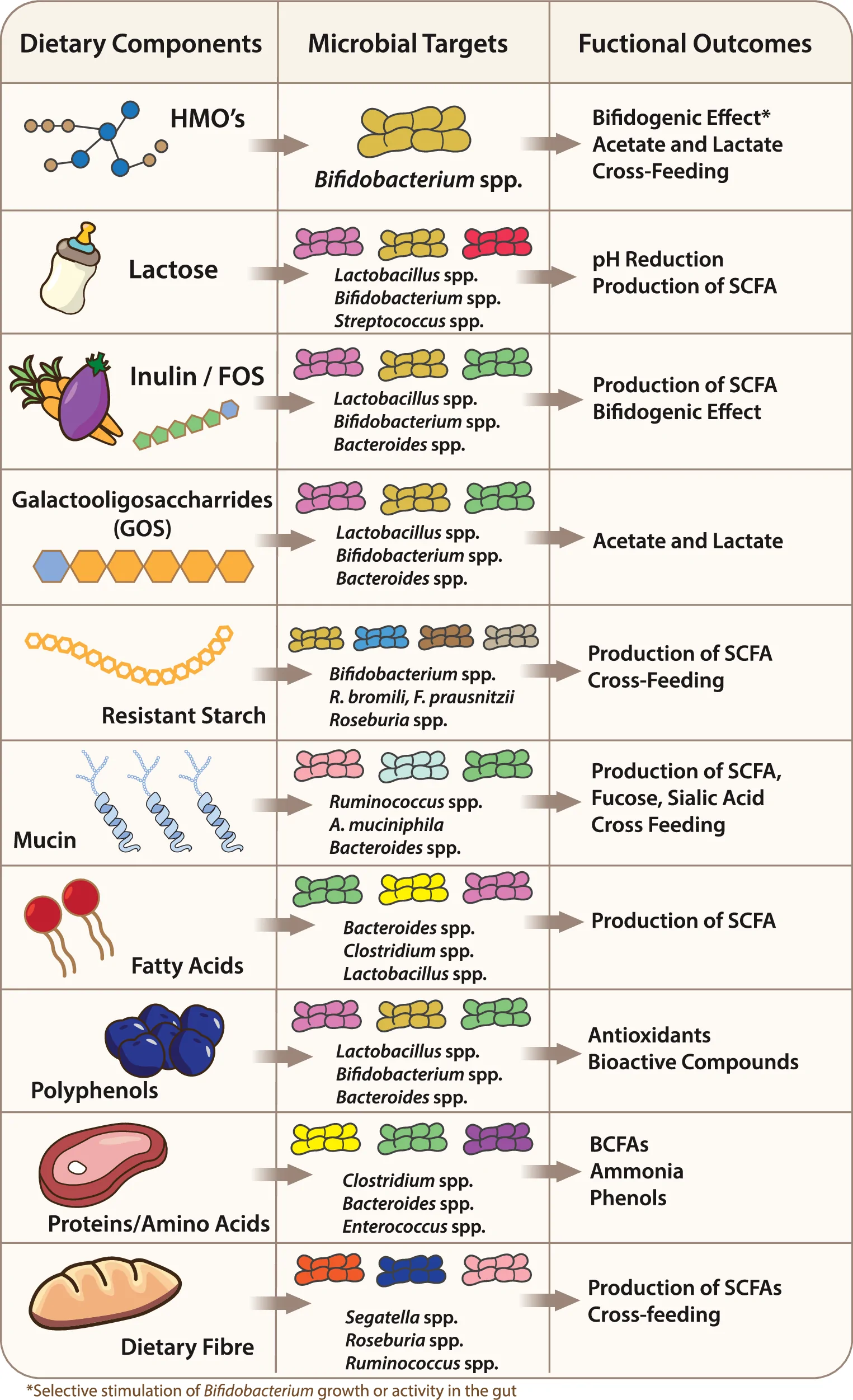
Dietary Input, Microbial targets and Functional outcomes^198–225^.

Diet is a major driver of gut microbial composition and function^72^. Fibre-rich substrates tend to promote saccharolytic communities and increased SCFA production (acetate, propionate, butyrate)^73–75^, whereas high-fat inputs can favour bile-tolerant and lipid-utilising taxa^76,77^. Protein-rich substrates often enrich amino acid fermenters and increase branched-chain fatty acid (BCFA) production^78–81^. SynComs provide a tractable platform to test these links mechanistically, connecting substrate availability to pathway activation, metabolite outputs, and downstream ecological outcomes. Key diet-target-function relationships are summarised in Fig. 3.

### Functional analysis of SynComs

Functional analysis is central to understanding how diet shapes SynComs. Moving beyond taxonomy, functional approaches reveal how communities metabolise substrates, produce key metabolites, and shift ecological roles. Nutrient-specific metabolism can directly alter the growth of individual strains and their competitive or cooperative interactions, ultimately influencing community structure, dominance and coexistence in complex SynComs^82^. Integrating multi-omics with targeted biochemical assays and computational tools enables more precise mapping of community function in response to dietary change (Table 2).

**Table 2:** Omic Approaches and their functional targets

Approach	Purpose	Functional Insight	References
Culturomics	High-throughput isolation and characterisation	Expands strain panels for SynComs	^124,179^
Metagenomics (Shotgun)	Taxonomic composition, gene content, functional gene potential (CAZymes, SCFA pathways, mucin-degrading enzymes)	Identifies microbial species present and their genetic potential for SCFA production, mucin and HMO degradation etc.	^28,180^
(Meta)Transcriptomics	Quantify strain level or Community gene wide expression to reveal active pathways at the time of sampling	Reveals which metabolic pathways are actively expressed (e.g., fucosidases, sialidases, SCFA synthesis genes)	^32^
Proteomics / Metaproteomics	Identify and quantity proteins and enzymes present in a sample	Validates functional expression of CAZymes, transporters, and enzymes for SCFA synthesis and mucin/HMO metabolism	^181–183^
Metabolomics	Targeted metabolomics quantify predefined metabolites (SCFAs, monosaccharides, organic acids, amino acids, bile acids); Untargeted metabolomics for broad profiling to detect known and novel metabolites.	Measures the products of microbial metabolism; quantifies SCFA profiles, mucin degradation products (fucose, sialic acid)	^89,184^
Lipidomics	Characterise lipid types and pathways to provide insight into microbe-lipid metabolism	Identification of bioactive lipids, phospholipids, sphingolipids, fatty acids etc.	^185,186^
Stable Isotope Probing	Incorporation of labelled substrates (e.g., ^13^C-HMOs) into microbial DNA/RNA/metabolites	Identifies microbes actively consuming specific substrates and tracks metabolic fluxes	^187–190^

### Application of SynComs in gut microbiota-diet research

SynComs are powerful tools for dissecting the complex interplay between diet, microbes and host health, because they move beyond largely observational diet-microbiome studies^83–87^, and allow controlled hypothesis-driven experiments. With defined strains and substrates, SynComs can directly test how specific dietary components are metabolised, how cross-feeding and competition shapes community structure, and how metabolite outputs link to host-relevant outcomes.

SynComs have been especially informative for diet-driven succession. For example, incorporating HMOs has shown how human milk selectively promotes *Bifidobacterium* spp. in early life^88^. Beyond this, SynCom-based models have been widely used to characterise microbial cross-feeding networks, revealing metabolic interdependencies and community assembly patterns in response to specific nutritional substrates^31,43,89–93^. Similarly, fibre and resistant starch SynComs have revealed how weaning-associated substrates drive diet-dependent ecological shifts, enriching saccharolytic taxa with specialised metabolic capacities and promoting SCFA production, ultimately contributing to microbiome maturation^43,94–97^.

As SynComs offer compositional control, they are also well-suited for mapping cooperative and competitive interactions^98^. This mechanistic resolution is valuable for studying diet-associated disease mechanisms, including malnutrition-related loss of diversity and metabolite production^99,100^ and Western diet-linked disruption of microbial metabolism and inflammation in inflammatory bowel disease (IBD) models^21,101–103^. Importantly, these controlled systems also direct interrogation of microbial metabolic outputs such as SCFAs, bile acid derivatives, or amino acid metabolites and host immune and metabolic responses^104–106^. More broadly, SynComs have been used to generate stronger mechanistic evidence connecting dietary perturbations to microbiota-mediated pathways implicated in metabolic and immune-mediated disorders, e.g. type 2 diabetes, obesity^107,108^ colorectal cancer^107^, allergy/asthma^109^, and in neurodevelopmental and neuropsychiatric outcomes^110^, helping to address ethical and practical limits of human studies.

SynComs also support personalised nutrition by enabling direct testing of inter-individual functional variation^111,112^. For instance, *Bifidobacterium* strains differ in their capacity to utilise specific HMOs, suggesting tailored probiotic or dietary approaches in infancy^43,113^. In adults, Community-Scale Metabolic Modelling (MCMM) and SynComs experiments show that microbial diversity drives distinct metabolite profiles under the same diet, reinforcing the rationale for precision nutrition^114–117^.

Finally, SynComs can function as intervention testbeds, allowing systematic evaluation of prebiotics, probiotics, and synbiotics, as well as how drugs are transformed by gut microbes and how microbial metabolism influences drug efficacy and toxicity^118–120^.

Together, these applications position SynComs as versatile, reproducible models that bridge diet, microbial ecology, and therapeutics to uncover mechanisms driving health and disease, including assessment of dietary intervention studies.

**Fig. 4:**
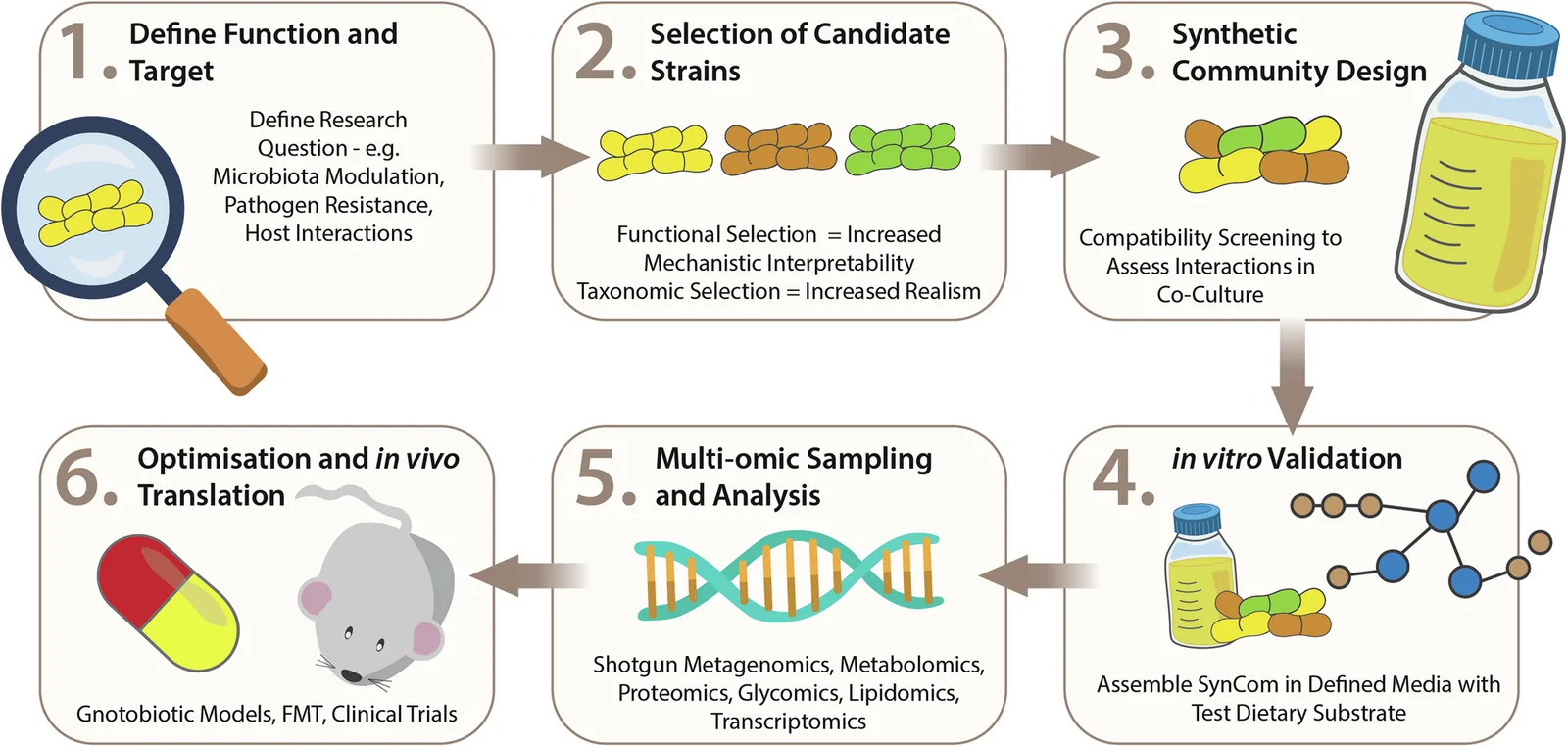
Workflow for SynComs-based dietary intervention study.

A structured overview of how these applications can be operationalised in practice is summarised in Fig. 4. In this framework, experimental development begins with defining the functional objective linked to a dietary substrate, thereby establishing the guiding research question. Candidate strains are then selected based on metabolic capacities relevant to the target substrate and their ability to occupy complementary metabolic niches while maintaining ecological relevance. The selected strains are assembled and evaluated for compatibility, including cooperative and competitive interactions that influence substrate utilisation and metabolite production. The SynCom is then tested in a controlled in vitro system containing the dietary substrate of interest to assess functional activity. Finally, multi‑omic analyses are applied to characterise community dynamics and quantify metabolite outputs, supporting optimisation and translation into in vivo or host‑relevant models.

Although presented in the context of diet-SynCom studies, the same underlying design principles are broadly applicable to other SynCom applications, including pathogen resistance, ecosystem function, and immune modulation, as the central logic of linking defined inputs to measurable functional outcomes remains consistent across experimental systems.

### Minimal reporting checklist for diet-based SynComs studies

Methodological transparency is essential in SynCom research to ensure reproducibility, comparability, and long-term utility^121^. Because SynComs experiments involve multiple design layers, from strain provenance, inoculum preparation, culture vessel operation, media composition, substrate characterisation, and multi-omics and analytical workflows, clear reporting is needed to minimise ambiguity. A tailored reporting checklist may help standardise documentation and support alignment with established reporting frameworks such as MIxS and STORMS^122,123^. We therefore propose a minimal checklist for diet-focused SynCom studies (Table 3) to harmonise reporting across experimental design, implementation, and analysis.

**Table 3:** Proposed Minimal Reporting Checklist for Diet-Based SynComs Studies

Category	Required Reporting Items
Strain Provenance and Identity	Strain names; accession IDs; Donor/Culture-collection source; authentication (Genome/ANI thresholds); antibiotic resistance profile where relevant
Genome characterisation	Assembly identifiers; version numbers; functional annotations (e.g., CAZymes, SCFA pathway genes); and links to public repositories (MIxS-compliant metadata).
Inoculum design	Complete strain list; initial abundances; revival procedures; passage history; viability checks; cryoprotectants, and co-adaptation steps (if any).
Culture Media and Anaerobic conditions	Full media composition (carbon/nitrogen sources; fibres/polymers), redox indicators, gas composition, pH set-points/controls.
Bioreactor parameters	Vessel type; operation mode; feed/wash rates (D); retention times, mucosal compartment (if present), temperature, agitation, sampling scheme; for SHIME/TIM-2 configuration citation.
Dietary Inputs	Substrate type; supplier; lot number; dosing schedule, and analytical verification (e.g., DP distribution, compositional profiling).
Controls and Reference standards	Sterile controls; negative controls; mock communities/standards used (e.g., NIBSC, commercial), spike-ins, and calibration curves
Multi-Omics and Analytics	Extraction kits/protocols; library preparation; sequencing platform; bioinformatics versions/parameters; STORMS/MIxS compliance; deposition of raw data + metadata in public repositories.
Host associated models	Facility SOPs; gnotobiotic monitoring; cage/isolator details; diet; colonisation verification; ethical approvals; references to established protocols or GM consortia where applicable.
Statistical considerations	Power calculations (where feasible); randomisation; batch handling; and pre-registered analyses for translational studies (where applicable).

### Challenges of using SynComs

SynComs are powerful, reductionist tools for dissecting diet-microbe, microbe-microbe, and host-microbe interactions under controlled conditions. However, wider uptake is still limited by several technical, ecological, and methodological challenges. Overcoming these will require coordinated innovation and greater standardisation to make SynComs more reproducible, scalable, and representative of the gut ecosystem.

#### Culturing and maintaining strict anaerobes

A major practical barrier is the cultivation and long-term maintenance of strict anaerobes, which comprise a large fraction of gut communities, yet are highly oxygen sensitive. Growth requires specialised anaerobic systems, oxygen-free media, and careful handling. Although culturomics has expanded the range of recovered gut anaerobes^22,124^, sustaining them within mixed communities remains difficult; viability can decline during extended in vitro culture, driving community drift and loss of diversity. This is particularly problematic for SynComs built around cross-feeding networks, where the loss of one anaerobe can disrupt dependent taxa and trigger cascading effects across the community, such as accumulation or depletion of critical metabolites and consequently destabilising dependent taxa. Such cascading effects can ultimately compromise the reproducibility, functionality, and ecological relevance of the SynCom, highlighting the need for improved strategies to preserve anaerobe viability and maintain community structure over time.

#### Selecting species that truly coexist

Designing stable SynComs requires careful selection of community members that can coexist without competitive exclusion. Strain incompatibility, including antagonism, resource competition, or unstable co-existence can arise in multiple ways depending on the isolates and experimental context^125–128^. Microbial interactions, such as substrate competition, bacteriocin production, and cross-feeding, are highly context dependent, hence difficult to define a universal framework for incompatibility^129–132^. Overlapping metabolic niches can destabilise communities, while some taxa depend on host-derived factors that are absent in vitro^21^. As a result, SynComs are often simplified around “robust growers” rather than ecological representativeness. Because interaction networks are difficult to predict a priori, assembly still frequently relies on trial-and-error, reducing reproducibility across studies. Emerging modelling frameworks can reduce this empiricism; tools such as Modelling and Inference of MICrobiomes Project (MIMIC)^133^ and Microbial COMmunity modelling (MICOM)^134^, use genome-scale reconstructions and ecological modelling to predict competition, cross-feeding potential and metabolic complementarity, enabling more rational pre-selection of compatible strains, thereby shifting from the trial-and-error assembly towards predictive, mechanism-informed design of SynComs.

#### Capturing gut complexity and resilience

The human gut microbiome comprises hundreds of interacting species embedded in dense ecological networks^135–137^. Even “complex” SynComs, typically containing 10–100 strains, cannot fully reproduce this taxonomic and functional breadth^138^. Rare taxa, sometimes important for resilience and redundancy, are frequently missing, limiting the ability of SynComs to reproduce ecological buffering, stability, and adaptability observed in vivo^131^.

#### Lack of standardisation across SynComs design

A key bottleneck is the absence of shared design and reporting frameworks. SynComs studies differ widely in strain sourcing, community composition, and culture conditions, often driven by availability rather than ecological rationale^21,32,139^. There is no consensus on the minimum number, breadth, or functional coverage required to “represent” a gut ecosystem^140^. Variation in media composition, inoculum preparation, and anaerobic handling further undermines comparability between studies. Establishing shared guidelines - analogous to the fields progress with standardised host-associated consortia and facility protocols (e.g., OMM12 and NGRRC), would substantially improve reproducibility and translational impact.

#### Limited long-term stability in vitro

In vitro SynComs often lose stability over time due to absence of host-derived ecological regulation. In the host, microbial communities are shaped by constant nutrient flow, mucosal secretions, immune regulation, and spatial structuring^141^. In vitro, even in continuous-flow fermenters, incomplete replication of these dynamics can lead to nutrient depletion, metabolite accumulation, and over-growth of fast-growing taxa^111^. While dynamic and continuous models extend stability, achieving long-term ecological balance and host-mediated feedback remains a key challenge.

### Future perspectives and emerging trends

Integrating SynCom experiments with multi-omics; genomics, transcriptomics, metabolomics, and proteomics is rapidly enhancing system-level mechanistic insight into microbial ecology. These approaches enable mapping of cross-feeding networks, functional redundancy, and metabolic fluxes under dietary perturbations^142^. Crucially, combined omics can link taxa to specific outputs (e.g., SCFAs or bile acid derivatives), helping explain not just who is present but what the community does and why it remains stable.

In parallel, advances in high-throughput culturomics, microfluidic isolation, and robotic automation are expanding the range of strains available for SynComs construction^143–146^. This is enabling larger communities with built-in redundancy that better capture native gut diversity and resilience. Coupling these pipelines to curated reference genome collections and defined strain libraries will further improve standardisation and sharing across laboratories.

A major translational step is the rise of hybrid microbe-host platforms, including microfluidic gut-on-a-chip systems and 2D/3D intestinal organoids, which allow SynCom co-culture with epithelial, and increasingly immune components^147^. As SynCom experiments can sometimes yield different functional outcomes in simplified in vitro systems compared to host-associated environments, where factors such as spatial structure, nutrient fluxes and immune responses shape microbial behaviour^28,148^, these models introduce physiological relevant gradients and mucosal interfaces, enabling controlled interrogation of diet-driven host-microbe interactions, barrier function, and immune signalling.

An emerging approach to building more realistic SynComs is to incorporate non-bacterial members of the gut ecosystem. Most SynComs still focus primarily on bacteria, but the gut ecosystem also includes fungi, viruses (predominantly bacteriophages), and archaea, all of which contribute to community assembly, stability, and host-microbe interactions^149–151^. Fungi can shape bacterial community structure through mechanisms such as nutrient competition and production of bioactive metabolites, and they can also influence host immune responses^152^, Similarly, bacteriophages can influence microbiome dynamics by targeting specific bacterial hosts and by lysogeny-associated effects (including gene transfer and altered bacterial phenotypes), thereby influencing bacterial abundance and function at the community level^151,153^. Archaea, especially methanogens, occupy a distinct metabolic niche and participate in syntrophic interactions (e.g., interspecies hydrogen transfer) that can influence bacterial fermentation and overall ecosystem energetics^154,155^. Building multi-kingdom SynComs is therefore an important step towards models that better reflect real gut ecology. However, there are substantial practical challenges, including differing growth requirements and replication times across kingdoms, the need to maintain stable multi-partner interactions under controlled conditions^149^, and the difficulty of incorporating phages whose host range is often narrow and highly context dependent, complicating reproducibility and predictive design^156^. Nevertheless, expanding SynComs beyond bacteria should improve ecological realism and mechanistic insights into community dynamics and intervention effects.

Another emerging direction is the use of ecological modelling and Artificial Intelligence (AI)-enabled design to replace trial-and-error community assembly with rational, predictive pipelines. Network, dynamical, and agent-based models can “forecast” stable configurations and functional complementarity^135,140,157,158^, while machine learning approaches mine large microbiome datasets to prioritise strain combinations and predict metabolic outputs^159–162^. Together, these developments promise more reproducible, and scalable SynCom design where communities are engineered to test explicit hypotheses about function, not simply to approximate diversity^159^.

Looking ahead, SynCom platforms are well positioned to support translational microbiome research by enabling the rational engineering and testing of microbiome-targeted therapeutics, including next-generation probiotics and biotherapeutics^163–165^, and by predicting host and microbial responses to dietary interventions, thereby informing personalised nutrition strategies^61,166,167^. In addition, SynComs enable mechanistic assessment of microbiome-drug interactions (including microbial metabolism of pharmaceuticals) and can serve as controlled preclinical platforms to de-risk interventions, identify functional biomarkers, and support precision nutrition and microbiome therapeutic strategies^168–174^.

## Conclusion

SynComs are powerful tools for untangling the complex interplay between diet and the gut microbiota. By reducing a naturally complex ecosystem into defined, controllable consortia, SynComs enable mechanistic tests of how dietary substrates influence ecology and metabolism, and how these microbial outputs influence host physiology^19,21^. Evidence from SynComs-based studies reinforces diet as a major driver of gut microbiota composition and function across the life course, from early colonisation through adulthood, with implications for long-term health^18,43^.

SynComs have generated key insights into nutrient specialisation, cross-feeding, succession, and diet-linked disease mechanisms, and critically, help bridge the gap between observational microbiome studies and causal inference by allowing diet-microbe-host hypotheses to be tested under controlled conditions^28^.

However, important challenges remain. These include cultivating and maintaining strict anaerobes, predicting stable coexistence, capturing the resilience and buffering capacity of native gut communities, and the lack of widely adopted frameworks for SynComs design and reporting. Many in vitro systems also have limited longevity, further limiting studies of long-term dynamics. Addressing these limitations will require continued advances in culturomics, improved continuous-culture platforms, and coordinated standardisation across laboratories.

Looking ahead, integrating SynComs with multi-omics, ecological modelling, multi-kingdom inclusion, and AI-driven design, alongside next-generation host-relevant platforms such as organoids and gut-on-a-chip systems, will enable ecological realism, reproducibility, and translational value. With these developments, SynComs research is well positioned to play a central role in building a more mechanistic, predictive understanding of diet-microbe-host research.

## Data Availability

No datasets were generated or analysed during the current study.
